# The deubiquitinase OTUD4 suppresses TAK1 kinase–dependent NF-κB signaling and inflammation

**DOI:** 10.1016/j.jbc.2025.110784

**Published:** 2025-10-07

**Authors:** Zhaohui Liu, Xiaolong Wang, Lihui Wu, Li Min, Ying Sheng, Zhiming Sun, Yunfang Deng, Lin Miao, Yue Liu, Jiabing Li, Yu Zhao

**Affiliations:** 1 The National & Local Joint Engineering Laboratory of Animal Peptide Drug Development, College of Life Sciences, Hunan Normal University, Changsha, Hunan, China; 2 Peptide and Small Molecule Drug R&D Platform, Furong Laboratory, Hunan Normal University, Changsha, Hunan, China; 3 Institute of Interdisciplinary Studies, Hunan Normal University, Changsha, Hunan, China

**Keywords:** NF-κB signaling, OTUD4, TAK1, ubiquitination, chronic inflammation

## Abstract

Chronic inflammation contributes to the development of many cancers, including non–small cell lung cancer, and is characterized by persistent activation of proinflammatory NF-κB signaling. The mechanisms that restrain NF-κB signaling remain incompletely defined. Here, we identify the deubiquitinase ovarian tumor family deubiquitinase 4 (OTUD4) as a suppressor of tumor necrosis factor (TNF)–induced NF-κB activation and chronic inflammation. OTUD4 interacts with core components of the transforming growth factor β–activated kinase 1 (TAK1) signalosome, including TAK1, TAB1, and TAB3, and removes K63-linked polyubiquitin chains from substrates within this complex, such as TAK1 and TAB3, thereby reducing TNF-induced NF-κB signaling. A histidine-centered loop (His loop) in the catalytic domain is required for this K63 linkage specificity. The tumor-associated OTUD4 H148Y missense variant (c.442C>T, *p.H148Y*), located within this loop, retains TAK1 binding but abolishes intrinsic deubiquitinase activity toward both K63- and K48-linked chains and is associated with sustained NF-κB activation and increased proinflammatory cytokine expression. Collectively, these results reveal a mechanism that suppresses TNF-induced NF-κB signaling and links OTUD4 dysfunction to inflammation-driven oncogenesis, including non–small cell lung cancer.

Chronic inflammation, a hallmark of cancer, fosters a protumorigenic microenvironment that fuels tumor growth and metastasis (1, 2, 3, 4). In many inflammation-driven cancers, such as non–small cell lung cancer (NSCLC), this state is driven by sustained activation of the NF-κB pathway (4, 5, 6). A pivotal node mediating this response is transforming growth factor β–activated kinase 1 (TAK1/MAP3K7), which is activated by cytokines, including tumor necrosis factor (TNF) (7). TAK1 activity is governed by a dynamic equilibrium: nondegradative K63-linked polyubiquitination activates the TAK1 signalosome *via* TAB adaptors, whereas deubiquitinases (DUBs) remove these activating chains (8, 9, 10, 11, 12, 13, 14, 15, 16, 17, 18). Disruption of this balance—for example, by loss-of-function variants in DUBs—has been implicated in the pathogenesis of inflammatory disease and cancer (19, 20, 21, 22, 23, 24).

The DUB ovarian tumor family deubiquitinase 4 (OTUD4), a member of the ovarian tumor (OTU) family, regulates diverse cellular processes through context-dependent mechanisms (25, 26, 27, 28, 29, 30, 31, 32). We previously showed two modes: (i) a noncatalytic scaffolding function that opposes K48-linked polyubiquitination to stabilize DNA alkylation repair enzymes (32) and (ii) an intrinsic, K63-directed catalytic activity that is activated from a dormant state by casein kinase 2–dependent phosphorylation and requires a ubiquitin (Ub)-interacting motif (UIM) for chain engagement, thereby attenuating MyD88-dependent signaling (31). Whether OTUD4 also regulates the TNF-triggered, MyD88-independent pathway has remained unclear. Although A20, CYLD, and OTUD5 limit TAK1 signaling through distinct mechanisms, the full complement of DUBs acting directly on the TAK1 signalosome remains unresolved (13, 15, 33). Motivated by our proteomic screen identifying TAK1 as a putative OTUD4 interactor (31), we asked whether OTUD4 acts directly at the TAK1 signalosome to regulate downstream signaling.

Pathogenic variants in DUBs are increasingly linked to dysregulated inflammatory signaling and cancer, yet the mechanistic links between genotype and pathway output remain poorly defined (19, 20, 21, 22, 23, 24). Of particular interest, the naturally occurring OTUD4 missense variant H148Y (c.442C>T, *p.H148Y*), identified in pheochromocytomas (34, 35, 36, 37, 38, 39, 40, 41, 42), resides within the catalytically essential His loop of the OTU domain (43, 44, 45), suggesting functional impairment. It remains unknown whether—and by what mechanisms—this clinically observed, structurally focal substitution perturbs OTUD4 catalytic activity or scaffold-dependent function, and whether any resulting changes influence TAK1-proximal inflammatory signaling.

Here, we identify OTUD4 as a direct suppressor of the TAK1 signalosome, a function that requires a catalytic His loop and is mediated by K63-linked deubiquitination. Using the tumor-associated H148Y variant, we demonstrate that this loop governs intrinsic catalysis but is dispensable for signalosome binding, thereby functionally decoupling these two key activities. Our results, therefore, reveal a new mechanism for attenuating TNF signaling and link loss of OTUD4 catalytic function to the proinflammatory states that can drive tumorigenesis.

## Results

### The tumor-associated OTUD4 H148Y variant abolishes intrinsic K63- and K48-linked DUB activity

**Figure 1:**
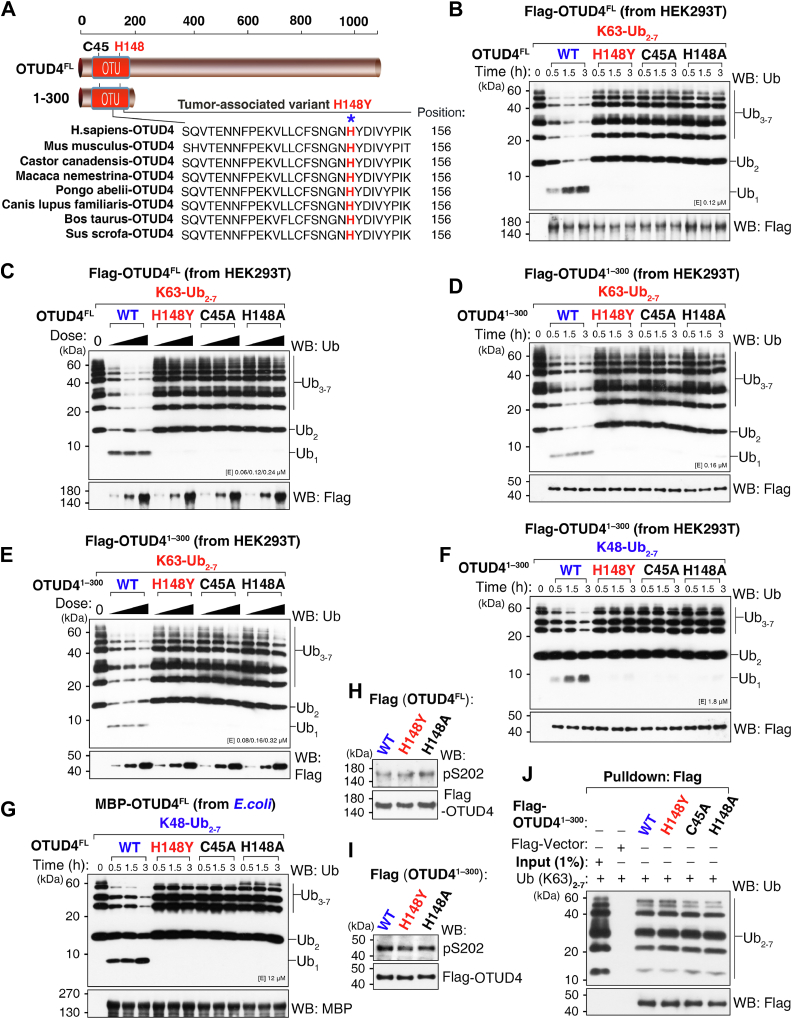
**The tumor-associated OTUD4 H148Y variant abolishes intrinsic K63- and K48-linked deubiquitinase activity.***A*, schematic of OTUD4 domains and sequence alignment of the conserved His loop across species; the catalytic histidine (H148) and tumor-associated H148Y are highlighted in *red*. *B*–*E*, *in vitro* deubiquitination assays were performed by incubating purified FLAG-tagged OTUD4 proteins (WT, H148Y, C45A, and H148A) with K63-linked polyubiquitin chains (Ub_2–7_); cleavage was assessed by Western blotting (WB) with an anti-Ub antibody. Time-course assays were performed with full-length OTUD4 (*B*) and OTUD4^1–300^ (amino acids [aa] 1 to 300) (*D*); dose–response assays were performed with full-length OTUD4 (*C*) and OTUD4^1–300^ (*E*). *F*, time-course deubiquitination of K48-linked polyubiquitin chains (Ub_2–7_) by OTUD4^1–300^ purified from HEK293T cells. Only WT displayed K48 cleavage, whereas H148Y, C45A, and H148A were inactive. *G*, time-course deubiquitination of K48-linked polyubiquitin chains (Ub_2–7_) by full-length MBP-tagged OTUD4 (WT, H148Y, C45A, and H148A) purified from *Escherichia coli*. *H* and *I*, WB analysis of Ser202 phosphorylation in full-length OTUD4 (*H*) and truncated OTUD4^1–300^ (*I*) (WT, H148Y, C45A, and H148A) using a pS202-specific antibody. *J*, *in vitro* pull-down assay assessing the interaction between OTUD4^1–300^ proteins (WT, H148Y, C45A, and H148A) and K63-linked poly-Ub chains (Ub_2–7_) in the presence of the deubiquitinase inhibitor *N*-ethylmaleimide (NEM, 10 mM); bound ubiquitin chains were detected by WB. Data are representative of at least three independent experiments. HEK293T, human embryonic kidney 293T cell line; MBP, maltose-binding protein; OTUD4, ovarian tumor family deubiquitinase 4; Ub, ubiquitin; WB, Western blot.

To assess the functional impact of a tumor-associated DUB variant, we examined the OTUD4 H148Y missense variant (c.442C>T, *p.H148Y*), cataloged in COSMIC (ID: COSP38343) and initially reported in pheochromocytoma (34). This substitution replaces a highly conserved histidine (H148) within the catalytic His loop of the OTU domain, a motif required for catalysis (Fig. 1*A*) (43, 44, 45). We generated full-length and catalytic domain–containing fragment (amino acids [aa] 1 to 300; hereafter OTUD4^1–300^) FLAG-tagged constructs of WT OTUD4 (WT), H148Y, C45A (active-site mutant), and H148A (histidine-to-alanine substitution control) and expressed them in human embryonic kidney 293T (HEK293T) cells. All proteins were purified to near homogeneity, and purity was verified by SDS-PAGE followed by silver staining (Fig. S1, *A* and *B*). In parallel, maltose-binding protein (MBP)–tagged full-length OTUD4 WT and variants were produced in *Escherichia coli* and purified for intrinsic activity assays (purity shown by SDS-PAGE and Coomassie staining; Fig. S1*C*).

To characterize enzymatic activity, we performed *in vitro* deubiquitination assays with purified full-length proteins and K63-linked di- to hepta-Ub_2–7_ chains, a well-characterized nondegradative linkage in inflammatory signaling (31). We monitored Ub chain cleavage over time and across enzyme concentrations. In both time-course and dose-dependent assays, WT OTUD4 efficiently cleaved K63-linked chains, whereas H148Y showed no detectable activity, similar to C45A and H148A (Fig. 1, *B* and *C*). A comparable activity loss was observed with OTUD4^1–300^, indicating that the defect maps to the catalytic core (Fig. 1, *D* and *E*).

We then examined whether the defect extends to K48 linkages. Using K48-linked di- to hepta-Ub_2–7_ chains as substrates, WT OTUD4 cleaved K48 polyubiquitin chains in assays using both OTUD4^1–300^ purified from HEK293T cells and MBP-OTUD4^FL^ purified from *E. coli*, whereas H148Y again showed no detectable activity, similar to C45A and H148A (Fig. 1, *F* and *G*). These data indicate that H148Y abolishes intrinsic DUB activity toward both K63 and K48 linkages and that the defect maps to the catalytic core.

Notably, when using full-length FLAG-OTUD4 proteins affinity purified from HEK293T cells, H148Y unexpectedly cleaved K48-linked Ub_2–7_ at WT levels; H148A and the active-site mutant C45A also showed K48 cleavage comparable to WT in both time-course and dose–response assays (Fig. S1, *D* and *E*). For C45A, this is consistent with prior rescue in the ALKBH3 model (32). All four OTUD4 proteins (WT, H148Y, H148A, and C45A) retained USP7 association by coimmunoprecipitation (co-IP) (Fig. S1*F*). Because intrinsic K48 activity was absent in both the OTUD4^1–300^ fragment purified from HEK293T cells (Fig. 1*F*), which lacks the DUB recruiting domain (DRD, aa 181–550) (32), and in full-length OTUD4 purified from *E. coli* (Fig. 1*G*), the K48 activity observed in full-length H148Y purified from HEK293T cells does not reflect intrinsic catalysis. Instead, this activity is mediated by copurifying cellular DUBs (*e.g.*, USP7) recruited *via* the intact scaffolding domain.

We next examined whether the catalytic defect in H148Y resulted from impaired regulatory inputs for OTUD4, specifically its phosphorylation at Ser202 (pS202-OTUD4) and its engagement with polyubiquitin chains *via* the UIM (31). Western blotting analysis of both full-length and N-terminal (aa 1–300) constructs showed comparable Ser202 phosphorylation in WT OTUD4, H148Y, and H148A, indicating that the catalytic defect is not attributable to altered Ser202 phosphorylation (Fig. 1, *H* and *I*). To assess Ub-chain binding independently of catalysis, we performed pull-down assays with K63-linked Ub_2–7_ in the presence of the DUB inhibitor *N*-ethylmaleimide. H148Y bound K63-linked Ub chains at WT levels; H148A and the active-site mutant C45A were similar, whereas the FLAG vector control showed no chain enrichment (Fig. 1*J*). Collectively, these results indicate that H148Y separates recruitment from catalysis: the variant retains Ser202 phosphorylation, UIM-mediated Ub-chain binding, and association with partner DUBs (*e.g.*, USP7), yet lacks intrinsic K63- and K48-linked DUB activity because of a specific defect within its catalytic His loop.

### OTUD4 interacts with the TAK1 signalosome

**Figure 2:**
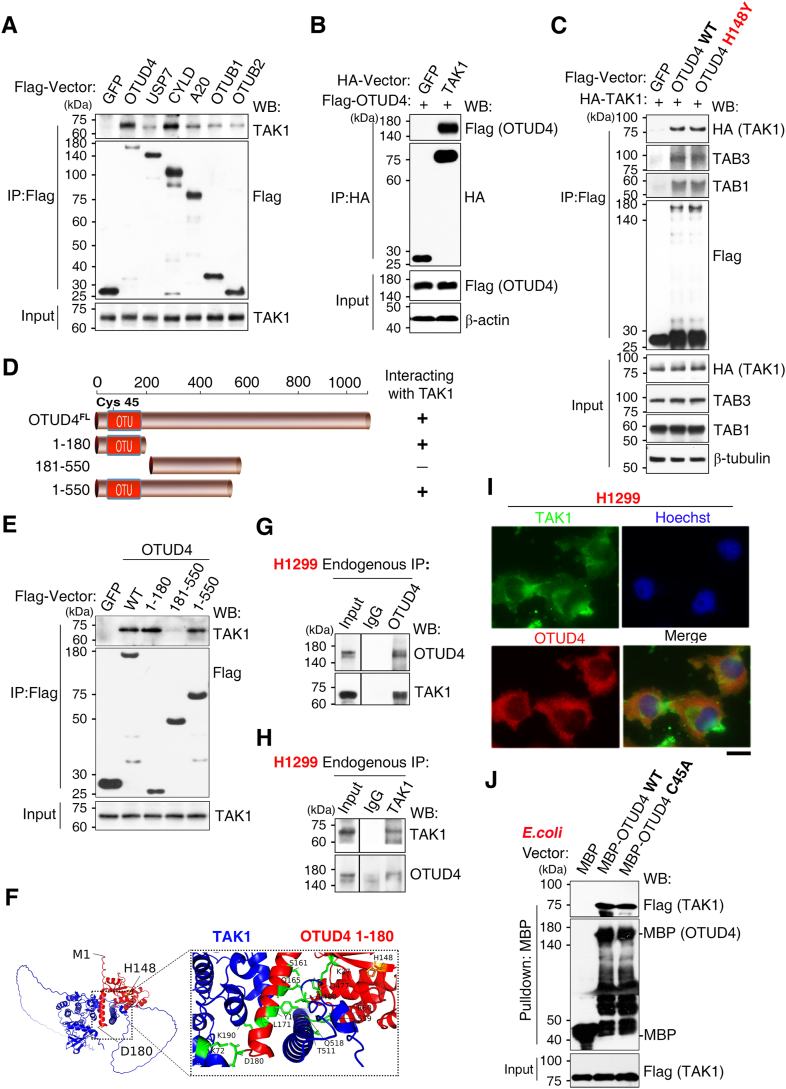
**OTUD4 interacts with the TAK1 signalosome.***A*, coimmunoprecipitation (co-IP) screen in HEK293T cells, analyzed by WB, identified a robust interaction between FLAG-OTUD4 and endogenous TAK1 among tested DUBs. *B*, this interaction was confirmed by reciprocal co-IP, where immunoprecipitation (IP) of HA-TAK1 coprecipitated FLAG-OTUD4. *C*, co-IP analysis showing that both FLAG-OTUD4 WT and the catalytically impaired OTUD4 H148Y variant associate with the complete TAK1 signalosome (HA-TAK1 together with endogenous TAB1 and TAB3). *D*, schematic of the OTUD4 deletion mutants used for interaction mapping. *E*, deletion mapping by co-IP identified the N-terminal OTU domain (aa 1–180) as necessary for binding TAK1. *F*, AlphaFold2 structural model of the predicted interface between the OTUD4 OTU domain (*red*) and TAK1 (*blue*). *G* and *H*, the endogenous OTUD4–TAK1 interaction in H1299 NSCLC cells was confirmed by reciprocal co-IP using antibodies to OTUD4 (*G*) and TAK1 (*H*). *I*, immunofluorescence (IF) staining of endogenous OTUD4 (*red*) and TAK1 (*green*) in H1299 cells shows cytoplasmic colocalization *(merge*); nuclei were counterstained with Hoechst (*blue*). The scale bar represents 20 μm. Quantification shows a positive correlation (PCC = 0.66 ± 0.15). *J*, direct interaction confirmed by an *in vitro* pull-down assay using purified recombinant MBP-OTUD4 and FLAG-TAK1 proteins. Data are representative of at least three independent experiments. DUB, deubiquitinase; HEK293T, human embryonic kidney 293T cell line; MBP, maltose-binding protein; OTUD4, ovarian tumor family deubiquitinase 4; PCC, Pearson's correlation coefficient; TAK1, transforming growth factor β–activated kinase 1; WB, Western blot.

Building on our previous proteomic analysis that identified TAK1 and its partners TAB1 and TAB3 as OTUD4 interactors (31) (Table S1), we performed co-IP assays in HEK293T cells to validate these findings. An initial screen of several DUBs confirmed a previously reported interaction between TAK1 and CYLD (15) but revealed that OTUD4 exhibited the most robust association (Fig. 2*A*). We prioritized the OTUD4–TAK1 interaction for further characterization.

Reciprocal co-IP confirmed this OTUD4–TAK1 association (Fig. 2*B*). OTUD4 also coimmunoprecipitated with the core signalosome components TAB1 and TAB3. Importantly, the intrinsically inactive H148Y variant bound the TAK1–TAB1–TAB3 complex with an efficiency comparable to that of WT OTUD4, indicating that the interaction is independent of OTUD4 catalytic activity (Fig. 2*C*). Furthermore, the assembly of the TAK1 signalosome remained intact, as the interaction between TAK1 and its endogenous partners TAB1 and TAB3 was unaffected by the presence or catalytic state of OTUD4 (Fig. S2*A*).

Deletion mapping experiments identified the N-terminal OTU domain (aa 1–180) as both necessary and sufficient for TAK1 binding (Fig. 2, *D* and *E*), a finding consistent with AlphaFold structural modeling predictions (Fig. 2*F*) (46). To confirm the physiological relevance of this interaction, we showed that endogenous OTUD4 and TAK1 associate in H1299 NSCLC cells (Fig. 2, *G* and *H*) and significantly colocalize in the cytoplasm (Pearson's correlation coefficient [PCC] = 0.66 ± 0.15; Fig. 2*I*). Finally, *in vitro* pull-down assays using purified recombinant proteins indicated that this interaction is direct (Fig. 2*J*). Collectively, these orthogonal experiments demonstrate a direct, cytoplasmic interaction between TAK1 and the N-terminal OTU domain of OTUD4.

### OTUD4 deubiquitinates TAK1 *via* K63-linked activity, a function abolished by the H148Y variant

**Figure 3:**
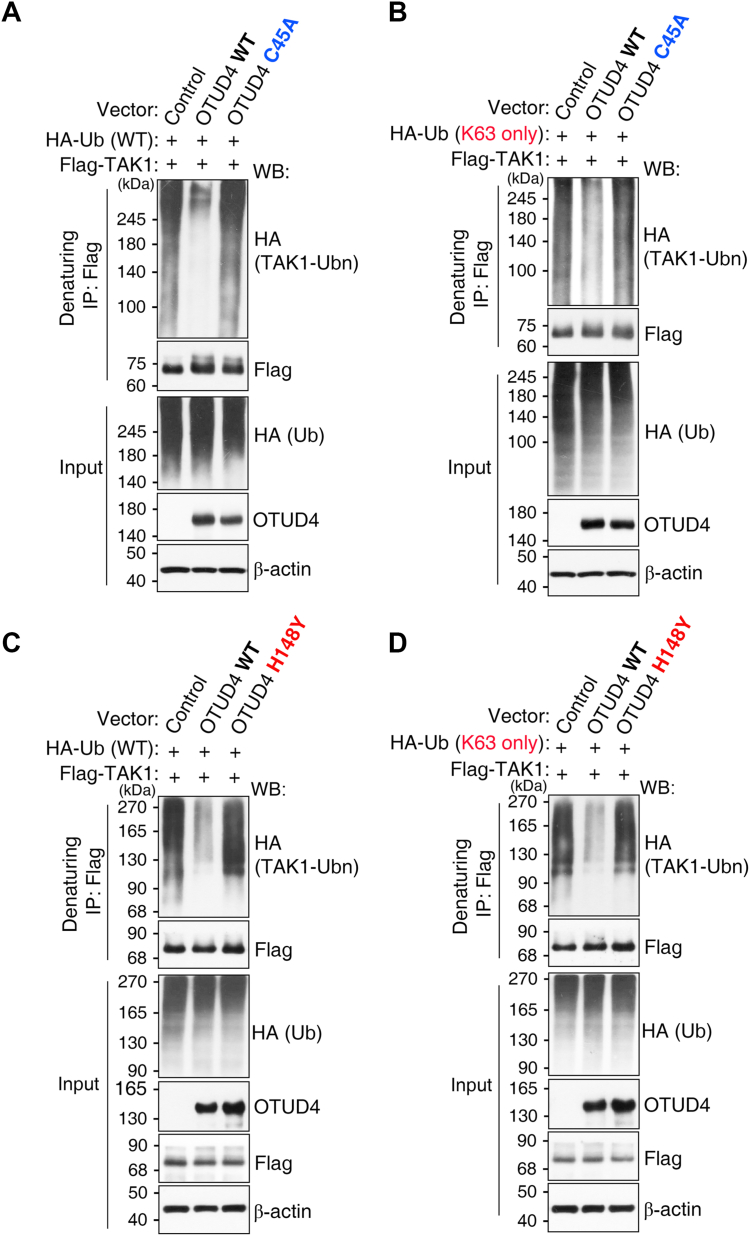
**OTUD4 deubiquitinates TAK1 with K63-linkage specificity.***A*–*D*, in-cell deubiquitination assays in HEK293T cells cotransfected with FLAG-TAK1, the indicated HA-ubiquitin (HA-Ub) constructs, and OTUD4 constructs; the ubiquitination status of FLAG-TAK1 was assessed by denaturing immunoprecipitation (IP) followed by WB with the indicated antibodies. *A*, coexpression of OTUD4 WT, but not catalytic mutant C45A, decreased total TAK1 ubiquitination. *B*, using a K63-only ubiquitin mutant, OTUD4 WT showed specificity for K63 linkages on TAK1. *C* and *D*, relative to OTUD4 WT, the tumor-associated H148Y variant showed impaired reduction of TAK1 ubiquitination with either HA-Ub (*C*) or K63-only HA-Ub (*D*). Data are representative of at least three independent experiments. HEK293T, human embryonic kidney 293T cell line; OTUD4, ovarian tumor family deubiquitinase 4; TAK1, transforming growth factor β–activated kinase 1; Ub, ubiquitin; WB, Western blot.

Given that OTUD4 directly binds the TAK1 signalosome, we next investigated whether OTUD4 deubiquitinates TAK1. To test this, FLAG-TAK1 and HA-Ub were coexpressed in HEK293T cells with either WT OTUD4 (WT) or the catalytically inactive C45A mutant. IP of TAK1 revealed that WT OTUD4, but not C45A, markedly reduced total TAK1 ubiquitination (Fig. 3*A*). To determine whether this activity was specific to the K63 linkages required for TAK1 activation, we repeated the assay using a K63-only Ub mutant. Indeed, WT OTUD4 substantially decreased K63-linked ubiquitination of TAK1, whereas C45A had no effect (Fig. 3*B*).

To assess whether intrinsic catalytic activity is required for this function, we tested the tumor-associated H148Y variant, which lacks intrinsic catalytic activity (Fig. 1). Consistent with this defect, H148Y failed to reduce total TAK1 ubiquitination (Fig. 3*C*) and, more specifically, was unable to remove K63-linked Ub chains (Fig. 3*D*). Together, these results demonstrate that OTUD4 suppresses TAK1 signaling by directly removing K63-linked polyubiquitin chains, a function requiring an intrinsic catalytic activity, mediated by the His loop, that is abolished by the tumor-associated H148Y variant.

### OTUD4 suppresses sustained TNF-induced NF-κB activation, a function impaired in the H148Y variant

**Figure 4:**
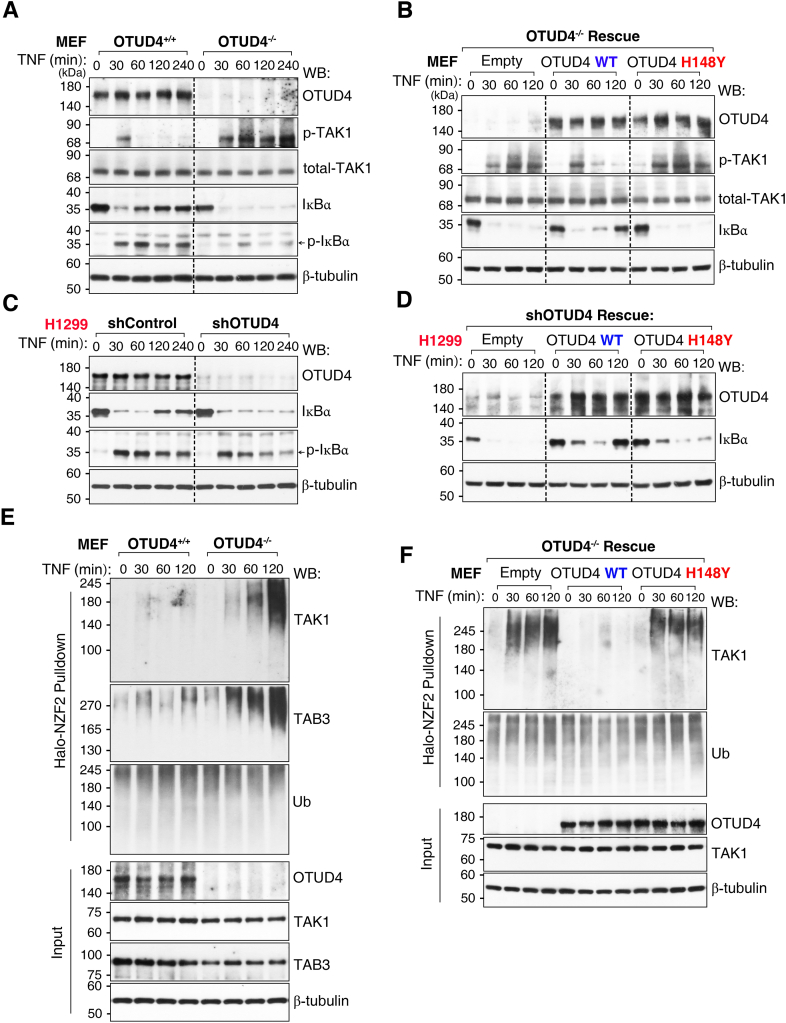
**OTUD4 suppresses sustained TNF-induced NF-κB activation, a function impaired in the H148Y variant.***A*, WT (OTUD4^+/+^) and OTUD4-knockout (OTUD4^−/−^) mouse embryonic fibroblasts (MEFs) were stimulated with TNF for the indicated times, and activation of the NF-κB pathway was analyzed by WB with the indicated antibodies. *B*, OTUD4^−/−^ MEFs were reconstituted with empty vector, OTUD4 WT, or the H148Y variant and analyzed as in (*A*). *C*, H1299 cells were transduced with control (shControl) or OTUD4-specific shRNA, stimulated with TNF, and analyzed by WB. *D*, the OTUD4-knockdown H1299 cells from (*C*) were reconstituted with empty vector, OTUD4 WT, or the H148Y variant and analyzed by WB with the indicated antibodies. *E*, K63-ubiquitinated proteins were enriched from lysates of TNF-stimulated WT and OTUD4^−/−^ MEFs under denaturing conditions using affinity purification with Halo-tagged NZF2 beads (Halo-NZF2 pull-down); bound proteins and total inputs were analyzed by WB with the indicated antibodies. *F*, Halo-NZF2 pull-down was performed using lysates from OTUD4^−/−^ MEFs reconstituted with an empty vector, OTUD4 WT, or the H148Y variant. Data are representative of at least three independent experiments. OTUD4, ovarian tumor family deubiquitinase 4; TNF, tumor necrosis factor; WB, Western blot.

To determine the functional consequence of the OTUD4–TAK1 interaction on downstream signaling, we stimulated WT (WT, OTUD4^+/+^) and OTUD4-knockout (OTUD4^−/−^) mouse embryonic fibroblasts (MEFs) with TNF. Compared with WT cells, OTUD4^−/−^ MEFs exhibited markedly prolonged phosphorylation of TAK1 and sustained degradation of the NF-κB inhibitor IκBα (Fig. 4*A*). The specificity of the p-TAK1 antibody was confirmed by phosphatase treatment (Fig. S2*B*).

To test the requirement for its intrinsic catalytic activity, we performed rescue experiments. Reintroduction of WT OTUD4 into OTUD4^−/−^ MEFs suppressed the prolonged TAK1 phosphorylation and restored IκBα turnover to basal levels. In contrast, the tumor-associated H148Y variant failed to rescue either phenotype (Fig. 4*B*). This regulatory mechanism was conserved in a disease-relevant context, as knockdown of endogenous OTUD4 in H1299 NSCLC cells reproduced the sustained IκBα degradation, which was rescued by re-expression of WT OTUD4 but not the H148Y variant (Fig. 4, *C* and *D*).

To link this signaling defect to the ubiquitination status of the TAK1 signalosome, we enriched K63-ubiquitinated proteins from TNF-stimulated MEFs using Halo-NZF2 affinity purification. OTUD4^−/−^ cells exhibited a pronounced and sustained increase in K63-linked ubiquitination of both TAK1 and its scaffolding partner TAB3 (Fig. 4*E*). This elevated ubiquitination was a direct consequence of the loss of OTUD4 catalytic activity, as it was reversed by the re-expression of WT OTUD4 but not the intrinsically inactive H148Y variant (Fig. 4*F*). Collectively, these results demonstrate that OTUD4 suppresses TNF-induced NF-κB signaling by removing K63-linked Ub chains from the TAK1 signalosome, a function that depends on intrinsic catalytic activity and is abolished by the tumor-associated H148Y variant.

### OTUD4 deficiency promotes sustained nuclear localization of p65 and inflammatory gene expression

**Figure 5:**
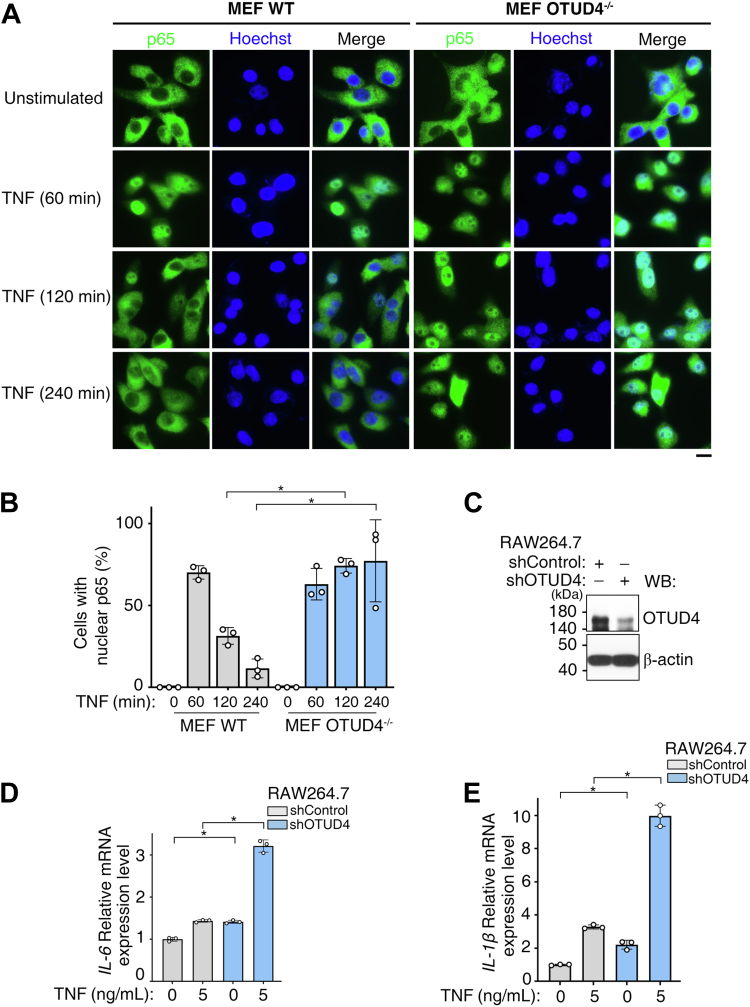
**OTUD4 deficiency promotes sustained nuclear localization of p65 and increased inflammatory gene expression.***A*, WT (OTUD4^+/+^) and OTUD4-knockout (OTUD4^−/−^) MEFs were stimulated with TNF for the indicated times, and the subcellular localization of p65 (*green*) was visualized by immunofluorescence (IF). Nuclei were counterstained with Hoechst (*blue*). The scale bar represents 20 μm. *B*, quantification of the percentage of cells with nuclear p65 from experiments as in (*A*). Data are presented as the mean ± SD from three independent experiments (n = 3, ∗*p* < 0.05). Each point represents one independent experiment, for which the percentage of cells with nuclear p65 was determined from at least 30 cells. *C*, WB analysis of OTUD4 protein levels in murine macrophage–like RAW 264.7 cells following lentiviral delivery of a nontargeting control shRNA (shControl) or an shRNA targeting OTUD4. β-actin served as a loading control. *D* and *E*, cells from (*C*) were stimulated with TNF (5 ng/ml), and relative mRNA levels of *IL-6* (*D*) and *IL-1β* (*E*) were measured by quantitative RT–PCR and normalized to β-actin. n = 3 biological replicates per group, and error bars indicate ±SD of the mean (∗*p* < 0.05). IL, interleukin; MEF, mouse embryonic fibroblast; OTUD4, ovarian tumor family deubiquitinase 4; TNF, tumor necrosis factor.

To determine the downstream consequences of persistent TAK1 activation, we assessed the nuclear translocation of the NF-κB subunit p65 in TNF-stimulated MEFs. Although TNF induced rapid p65 nuclear import in both WT and OTUD4^−/−^ cells, p65 was exported back to the cytoplasm in WT cells by 120 min, whereas it persisted in the nucleus of OTUD4^−/−^ cells (Fig. 5*A*). Quantitative analysis confirmed a higher proportion of OTUD4^−/−^ cells with nuclear p65 at later time points (Fig. 5*B*; *p* < 0.05). In a more immunologically relevant model, shRNA-mediated knockdown of OTUD4 in murine macrophage–like RAW 264.7 cells (Fig. 5*C*) led to a sustained upregulation of the proinflammatory cytokine genes *IL-6* (interleukin-6) and *IL-1β* following TNF stimulation, as measured by quantitative RT–PCR (qRT–PCR) (Fig. 5, *D* and *E*; *p* < 0.05). Collectively, these results indicate that the loss of OTUD4 prevents the timely attenuation of TAK1 signaling, resulting in prolonged p65 nuclear localization and an enhanced proinflammatory transcriptional response.

### The H148Y variant sustains NF-κB activation and exacerbates inflammatory responses

**Figure 6:**
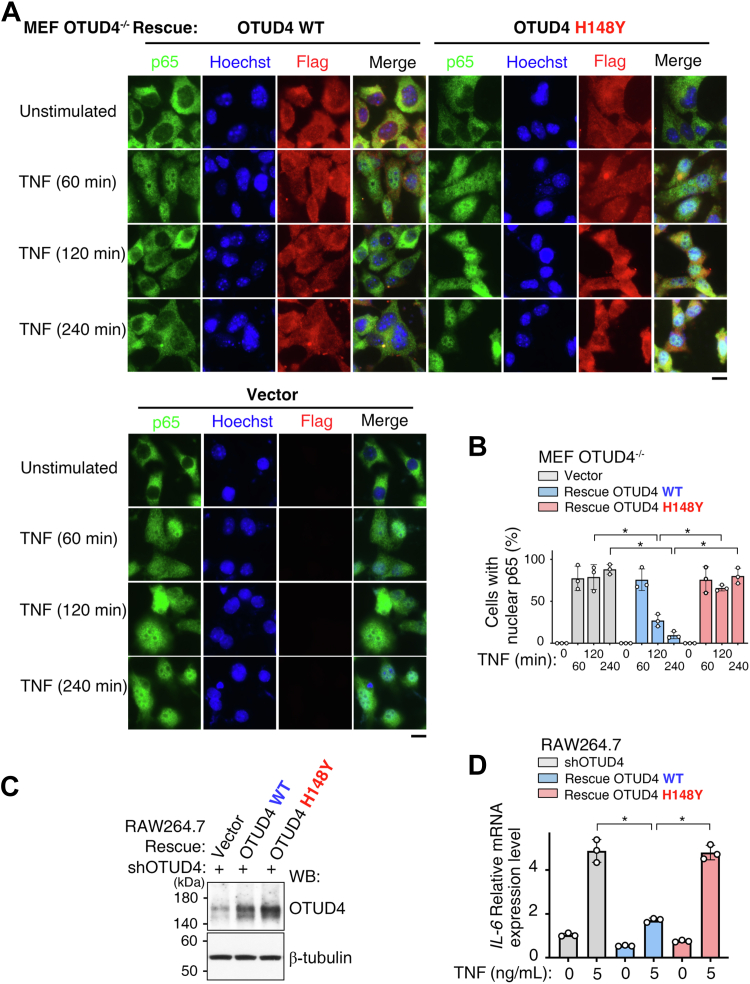
**The tumor-associated OTUD4 H148Y variant sustains NF-κB activation and exacerbates inflammatory responses.***A*, OTUD4-knockout (OTUD4^−/−^) MEFs were reconstituted with empty vector, OTUD4 WT, or H148Y variant. Following stimulation with TNF for the indicated times, the subcellular localization of p65 (*green*) was visualized by immunofluorescence (IF), with Hoechst (*blue*) as a nuclear counterstain. The scale bar represents 20 μm. *B*, quantification of the percentage of cells with nuclear p65 from experiments as in (*A*). Data are presented as the mean ± SD from three independent experiments (n = 3, ∗*p* < 0.05). Each point represents one independent experiment, for which the percentage of cells with nuclear p65 was determined from at least 40 cells. *C*, WB analysis of rescue experiments in RAW 264.7 cells. Endogenous OTUD4 was first depleted *via* lentiviral shRNA, and the shRNA-resistant rescue constructs (OTUD4 WT and H148Y) were subsequently introduced *via* a second lentiviral transduction. β-tubulin serves as a loading control. *D*, the reconstituted cells from (*C*) were stimulated with TNF (5 ng/ml), and relative *IL-6* mRNA levels were measured by quantitative RT–PCR and normalized to β-actin. n = 3 biological replicates per group, and the error bars indicate ±SD of the mean (∗*p* < 0.05). IL, interleukin; MEF, mouse embryonic fibroblast; OTUD4, ovarian tumor family deubiquitinase 4; TNF, tumor necrosis factor.

To determine the functional consequences of the H148Y variant, we performed rescue experiments in OTUD4^−/−^ MEFs. While re-expression of WT OTUD4 restored normal p65 nuclear export following TNF stimulation, the H148Y variant failed to do so, resulting in sustained p65 nuclear accumulation (Fig. 6, *A* and *B*; *p* < 0.05). To evaluate the transcriptional consequence of this signaling defect, we performed similar rescue experiments in OTUD4-depleted RAW 264.7 cells (Fig. 6*C*). Consistent with the p65 localization data, re-expression of the H148Y variant failed to suppress the TNF-induced expression of *IL-6*, in contrast to the rescue observed with WT OTUD4 (Fig. 6*D*; *p* < 0.05). Collectively, these findings indicate that the loss of intrinsic catalytic activity in the tumor-associated H148Y variant leads to a sustained NF-κB response and increased proinflammatory gene expression.

## Discussion

We previously showed that OTUD4 functions *via* distinct mechanisms: a noncatalytic scaffolding activity that stabilizes alkylation repair enzymes and a phosphorylation-activated, K63-directed catalytic activity that attenuates MyD88-dependent acute inflammation (31, 32). Building on this mechanistic diversity, we now identify a third mode in which OTUD4 suppresses TNF signaling *via* direct engagement of the TAK1 signalosome. This activity requires a histidine-centered loop (His loop) in the catalytic domain. Disruption of this loop, modeled by the tumor-associated H148Y variant, abolishes intrinsic activity toward both K63- and K48-linked chains, thus providing a direct link between OTUD4 catalytic failure and persistent proinflammatory signaling.

A key mechanistic insight from our study is that the His loop plays a critical role in regulating OTUD4 catalytic activity. Two complementary substitutions at H148—the tumor-associated H148Y variant and the histidine-to-alanine control H148A—provide key evidence for this mechanism. These substitutions did not affect UIM-mediated chain binding (Fig. 1*J*) or Ser202 phosphorylation (Fig. 1, *H* and *I*), two inputs previously shown to support activity toward K63-linked chains (31). Despite these functions remaining intact, catalytic output was markedly impaired. Specifically, H148Y abolished intrinsic activity toward K63-linked polyubiquitin chains in both full-length OTUD4 and OTUD4^1–300^, and H148A produced a comparable loss (Fig. 1, *B*–*E*). For K48-linked polyubiquitin chains, H148Y eliminated intrinsic activity in OTUD4^1–300^, and H148A produced a similar loss (Fig. 1*F*). In full-length OTUD4 expressed in *E. coli*, both substitutions markedly reduced intrinsic activity (Fig. 1*G*). Together, these results indicate that the His loop, disrupted by the tumor-associated H148Y variant, functions independently of UIM binding and Ser202 phosphorylation to regulate intrinsic OTUD4 catalytic activity across linkage types.

Intriguingly, although the OTUD4^1–300^ fragment was catalytically inert, the full-length H148Y purified from HEK293T cells retained detectable activity toward K48-linked polyubiquitin chains (Fig. S1, *D* and *E*). This paradoxical readout reflects scaffolding rather than intrinsic catalysis: the full-length OTUD4 contains a central recruitment domain (DRD, aa 181–550) absent from OTUD4^1–300^ (32). Consistent with a scaffold-dependent mechanism, the OTUD4 C45A catalytic mutant showed the same K48 readout. Like the H148 variants, C45A retained interaction with USP7, a well-characterized DUB that removes K48-linked polyubiquitin chains from multiple substrates (32, 47, 48) (Fig. S1*F*). Together, these results indicate that the K48 activity observed in full-length OTUD4 H148Y, H148A, and C45A is mediated by DRD-dependent recruitment of cellular DUBs (*e.g.*, USP7), rather than intrinsic OTUD4 catalysis. Thus, intrinsic OTUD4 catalysis, gated by the His loop, is functionally decoupled from DRD-supported scaffolding.

Beyond its previously reported role in upstream IL-1β–Toll-like receptor signaling (31), we now show that OTUD4 suppresses the TNF signaling by deubiquitinating the TAK1 signalosome. OTUD4 bound TAK1 directly and coimmunoprecipitated with TAB1 and TAB3 (Fig. 2, *A–C*, and *J*). Although OTUD4 efficiently removed K63-linked polyubiquitin chains from core components like TAK1 and TAB3 (Figs. 3 and 4*E*), the TAK1 signalosome assembly remained fully intact; its core scaffold interactions were stable and unaffected by the presence or catalytic state of OTUD4 (Fig. S2*A*). These findings support a model in which OTUD4 attenuates signaling by acting on substrates within a preassembled complex, rather than by triggering its disassembly. This mechanism differs from those reported for other DUBs like CYLD, A20 (15, 33), and is distinct from that of OTUD5, which inhibits TAK1 by promoting scaffold dissociation (13).

Dissecting this unique catalytic mechanism provides a framework for understanding how OTUD4 dysfunction contributes to inflammation-associated cancers such as NSCLC. To probe this link, we used the naturally occurring H148Y substitution—identified in pheochromocytoma (34)—as a molecular tool. The H148Y substitution is particularly informative because it functionally separates intrinsic catalysis that depends on the His loop from DRD-supported scaffolding, enabling isolation of the specific contribution of removal of K63-linked polyubiquitin chains to signal resolution (Fig. 1). Although H148Y has not been reported in NSCLC, in which chronic TNF-driven TAK1 activation is implicated (4, 5, 6, 49), these data support a model in which other loss-of-function OTUD4 variants impair intrinsic catalysis and maintain a proinflammatory state that promotes tumorigenesis.

In conclusion, this study identifies a regulatory node in the NF-κB pathway: OTUD4 suppresses inflammation by directly deubiquitinating K63-linked polyubiquitin chains from components of the TAK1 signalosome. This function requires a catalytic His loop and is disrupted by the tumor-associated H148Y substitution, linking impaired OTUD4 catalysis to persistent proinflammatory signaling. The translational relevance of these findings, however, remains to be determined, as the scope of our study was limited to cellular systems and a variant that is not yet clinically associated with NSCLC. Future studies should aim to validate these mechanisms *in vivo* and to identify and characterize loss-of-function OTUD4 variants in NSCLC and other patient cohorts. Such work will be critical to connecting OTUD4 dysfunction to cancer progression and to informing therapeutic strategies.

## Experimental procedures

### Cell culture

Human H1299 cells were purchased from the Cell Bank of the Chinese Academy of Sciences. HEK293T cells were obtained from the American Type Culture Collection, and murine macrophage–like RAW 264.7 cells were purchased from Procell Life Science & Technology Co, Ltd. Cells were cultured in Dulbecco’s modified Eagle’s medium (10313039; Gibco) supplemented with 10% heat-inactivated fetal bovine serum (10099-141C; Gibco) and penicillin–streptomycin (15140122; Gibco) at 37 °C in a humidified incubator with 5% CO_2_. All experiments used low-passage cells (between passages 5 and 15) to ensure experimental consistency and reproducibility. Routine mycoplasma testing was conducted using the Myco-Lumi Luminescent Mycoplasma Detection Kit (Beyotime) to ensure the absence of contamination.

### Antibodies

The anti-MBP rabbit antibody (E8030S; New England Biolabs [NEB]) was purchased from NEB. Antibodies against OTUD4 (25070-1-AP), TAK1 (12330-2-AP), and HA (51064-2-AP), as well as horseradish peroxidase (HRP)–conjugated anti-rabbit IgG (SA00001-2) and anti-mouse IgG (SA00001-1), were purchased from Proteintech. Antibodies against phospho-TAK1 (4536), p65 (8242), IκBα (9242), and phospho-IκBα (9246) were obtained from Cell Signaling Technology. Anti-Ub (sc-8017; Santa Cruz Biotechnology) and anti-FLAG M2 (F3165; Sigma–Aldrich) were obtained from Santa Cruz Biotechnology and Sigma–Aldrich, respectively. HRP-conjugated anti-β-actin (A00730; GenScript) and anti-β-tubulin (E021043; EarthOx) were loading controls. Secondary antibodies for immunofluorescence were Alexa Fluor 488–conjugated goat anti-mouse IgG (H + L) (A-11029) and Alexa Fluor 594–conjugated goat anti-rabbit IgG (H + L) (A-11037), both from Thermo Fisher Scientific.

### Plasmid constructs, transfection, and lentiviral infection

Full-length human OTUD4 complementary DNA (cDNA), as previously described (32), was used as the template for generating mutant constructs. Site-directed mutagenesis was performed to generate OTUD4 variants, which were then cloned into either pENTR 3C or pENTR 4 vectors (Invitrogen) using standard PCR protocols. Gateway LR recombination was used to transfer the inserts into pHAGE-FLAG or pHAGE-HA destination vectors. For bacterial expression in *E. coli*, the corresponding OTUD4 fragment was cloned into a pET-28a-FLAG vector with a FLAG tag or a pMAL-c5E vector with an MBP tag. The integrity of all PCR-derived constructs, including deletion and point mutation variants, was confirmed by Sanger sequencing.

To generate shRNA-resistant forms of OTUD4 WT, C45A, H148Y, and H148A, eight synonymous mutations were introduced into the region of the OTUD4 ORF targeted by the shRNA. Transient transfections were carried out using Lipo8000 Transfection Reagent (C0533; Beyotime) according to the manufacturer's protocol. Lentiviral particles were produced by transfecting HEK293T cells with pLKO.1 or pHAGE vectors and packaging plasmids. Viral supernatants were collected 72 h post-transfection and filtered through a 0.45 μm membrane. Target cells were transduced with filtered viral supernatant in the presence of 6 μg/ml polybrene for 12 h. After medium replacement, stable cell lines were selected using blasticidin or puromycin. The shRNA knockdown efficiency was confirmed by Western blotting.

### Purification of recombinant OTUD4 proteins for in vitro DUB activity assays

To purify OTUD4 proteins from eukaryotic cells, HEK293T cells stably expressing FLAG-tagged full-length OTUD4 (WT, H148Y, H148A, or C45A) or truncated OTUD4^1–300^ (WT, H148Y, H148A, or C45A) were lysed in lysis buffer (150 mM NaCl, 50 mM Tris–HCl, pH 7.9, 1% Triton X-100, 10% glycerol, and 2 mM DTT) supplemented with protease and phosphatase inhibitors (P1051; Beyotime). Clarified lysates were incubated overnight at 4 °C with anti-FLAG M2-agarose beads (A2220; Sigma–Aldrich). Beads were washed five times with the same lysis buffer. Bound proteins were eluted with 150 ng/μl FLAG peptide (F3290; Sigma–Aldrich) prepared in Tris-buffered saline (TBS). Eluted proteins were analyzed by SDS-PAGE and silver staining (Fast Silver Stain Kit; Sangon Biotech).

To obtain OTUD4 proteins from a prokaryotic system, full-length MBP-tagged OTUD4 (WT, H148Y, H148A, or C45A) was expressed in *E. coli* Rosetta (DE3) (Merck). Cultures were grown at 37 °C to an absorbance of 0.8 at 600 nm, and protein expression was induced with 0.7 mM IPTG at 16 °C overnight. Cells were resuspended in lysis buffer (150 mM NaCl, 50 mM Tris–HCl, pH 7.9, 1% Triton X-100, 10% glycerol, and 2 mM DTT) supplemented with protease and phosphatase inhibitors (P1031; Beyotime) and lysed by sonication. The clarified lysate was incubated with amylose resin (E8021S; NEB). Bound proteins were eluted with elution buffer (150 mM NaCl, 50 mM Tris–HCl, pH 7.9, 5% glycerol, and 2 mM DTT) supplemented with 20 mM maltose (A363021; Sangon Biotech). Eluted proteins were assessed by SDS-PAGE and Coomassie Brilliant Blue staining.

For *in vitro* DUB activity assays, purified recombinant proteins were incubated at 37 °C with K63-linked polyubiquitin chains Ub_2–7_ (UC-330; Boston Biochem) or K48-linked polyubiquitin chains Ub_2–7_ (UC-230; Boston Biochem) in DUB assay buffer (150 mM NaCl, 50 mM Tris–HCl, pH 7.9, 5% glycerol, and 2 mM DTT) for the indicated times. DUB activity was evaluated by Western blotting using an anti-Ub antibody, based on the appearance of free Ub or a reduction in polyubiquitin chain length.

### IP and Western blotting

IP assays were performed as previously described (31). Briefly, HEK293T cells were transfected with the indicated expression plasmids using Lipo8000 Transfection Reagent (C0533; Beyotime) and cultured for 48 to 72 h. Cells were harvested and lysed in lysis buffer supplemented with protease and phosphatase inhibitors. Lysates were clarified by centrifugation and incubated overnight at 4 °C with anti-FLAG M2-agarose beads (A2220; Sigma–Aldrich) or anti-HA-agarose beads (SC-7392AC; Santa Cruz Biotechnology) with gentle rotation. After washing with wash buffer, bound proteins were eluted by boiling in Laemmli sample buffer and analyzed by Western blotting.

For denaturing IP, lysates were boiled in TBS containing 1% SDS and diluted with lysis buffer to a final SDS concentration of 0.1% before IP. Eluted proteins were analyzed by Western blotting.

For Western blotting, protein samples were resolved by SDS-PAGE and transferred onto polyvinylidene difluoride membranes (Millipore). Membranes were blocked and incubated with primary antibodies, followed by HRP-conjugated secondary antibodies. Protein bands were detected using enhanced chemiluminescence.

### MBP pull-down assay

MBP-tagged OTUD4 constructs, MBP alone, and FLAG-tagged TAK1, all expressed in *E. coli* Rosetta (DE3) (Merck), were used for the pull-down assay. Bacterial cultures containing the respective expression plasmids (MBP-OTUD4 constructs, MBP alone, or FLAG-TAK1) were grown at 37 °C to an absorbance of 0.8 at 600 nm. Protein expression was induced with 0.7 mM IPTG, and cultures were incubated overnight at 16 °C. Cells were harvested by centrifugation at 8000*g* for 15 min at 4 °C and resuspended in lysis buffer as described earlier. Lysates containing MBP-OTUD4 or MBP alone were incubated with amylose resin (E8021S; NEB) at 4 °C for 12 h under gentle rotation to bind the fusion proteins. After three washes with wash buffer (as described earlier), the resin was incubated with bacterial lysates containing FLAG-TAK1 at 4 °C for 2 h under gentle rotation. Following three additional washes with wash buffer, bound proteins were eluted by boiling in Laemmli sample buffer and analyzed by Western blotting.

### Immunofluorescence assay

Cells were plated onto sterile round glass coverslips placed in 12-well plates. After attachment, cells were fixed with 4% paraformaldehyde at room temperature for 30 min, followed by permeabilization with 0.5% Triton X-100 in PBS for 20 min. Cells were washed three times with TBST, blocked, and incubated overnight at 4 °C with primary antibodies, with gentle agitation. The following day, coverslips were washed four times with TBST and incubated with appropriate secondary antibodies and Hoechst 33342 (561908; BD Biosciences) for 1 h at 37 °C in the dark. After the final washes, coverslips were mounted in antifade mounting medium, and fluorescence images were acquired using a fluorescence microscope (Olympus). The PCC was used to assess the colocalization between TAK1 and OTUD4. PCC values were calculated from 42 cells pooled from two biologically independent experiments (n = 42). The sample size was determined based on common practice in image-based colocalization studies; no formal power analysis was performed.

### RNA extraction and qRT–PCR

Total RNA was extracted using the RNAeasy RNA Isolation Kit (R0026; Beyotime) according to the manufacturer’s instructions. RNA concentration and purity were determined using a NanoDrop spectrophotometer (Thermo Fisher Scientific) by measuring absorbance at 260 nm and calculating absorbance at 260 nm/280 nm and absorbance at 260 nm/230 nm ratios. First-strand cDNA synthesis was performed using a BeyoFast cDNA Synthesis Kit (D7180M; Beyotime), followed by qPCR using the BeyoFast SYBR Green qPCR Mix (D7260; Beyotime) according to the manufacturer’s instructions. Gene expression levels were normalized to β-actin as an internal control. The following primer sequences were used for qRT–PCR: *IL-1β*, forward primer, 5′-TTC AGG CAG GCA GTA TCA CTC-3′, and reverse primer, 5′-GAA GGT CCA CGG GAA AGA CAC-3′; *IL-6*, forward primer, 5′-TAG TCC TTC CTA CCC CAA TTT CC-3′, and reverse primer, 5′-TTG GTC CTT AGC CAC TCC TTC-3′; and *β-actin*, forward primer, 5′-TCA TCA CTA TTG GCA ACG AGC GGT TC-3′, and reverse primer, 5′-TAC CAC CAG ACA GCA CTG TGT TGG CA-3′.

### Affinity purification of K63-linked polyubiquitinated proteins using Halo-NZF2

K63-linked polyubiquitin chains and their associated proteins were purified from cell lysates using a Halo-NZF2 affinity purification method, as previously described (31). Briefly, cells were collected following TNF stimulation, lysed, and denatured by boiling in TBS containing 1% SDS. Lysates were diluted with lysis buffer to a final SDS concentration of 0.1% before affinity purification. Cell extracts were incubated overnight at 4 °C with 10 μl of HaloLink resin (G1915; Promega) prebound to purified Halo-NZF2 protein (7 μg/sample) under gentle rotation. After incubation, the resin was washed five times with wash buffer, and bound proteins were eluted by boiling in Laemmli sample buffer. Eluted proteins were subjected to Western blotting analysis.

### Quantification and statistical analysis

Data are presented as mean ± SD from at least three independent experiments. Statistical analyses were conducted using GraphPad Prism, version 7.0 (GraphPad Software). Statistical significance was assessed using Student's *t* test or one-way ANOVA, as appropriate, with *p* values <0.05 considered significant (∗*p* < 0.05).

## Data availability

All data supporting the results of this study are available within the article and its supplementary information.

## Supporting information

This article contains supporting information.

## Ethics statement

This study did not involve any experiments with animals or human participants.

## Conflict of interest

The authors declare that they have no conflicts of interest with the contents of this article.
