# Supplementary material for: The deubiquitinase OTUD4 suppresses TAK1 kinase–dependent NF-κB signaling and inflammation
Source: J Biol Chem. 2025 Oct 7;301(11):110784. doi: 10.1016/j.jbc.2025.110784 (PMC12607013; doi:10.1016/j.jbc.2025.110784)
Supplement: Supplementary Figure Legends [file mmc1.docx]

**Supplementary Figure Legends**

**Supplementary Figure S1.** **Characterization of purified OTUD4 proteins and their interactions.** *A* and *B*, Silver-stained SDS-PAGE gels show the purity of various FLAG-tagged OTUD4 proteins purified from HEK293T cells. The gels display wild-type (WT), H148Y, C45A, and H148A variants of full-length OTUD4^FL^ in (*A*) and truncated OTUD4 (OTUD4^1–300^) in (*B*). *C*, Coomassie Brilliant Blue-stained SDS-PAGE gel of MBP-tagged full-length OTUD4 (MBP-OTUD4^FL^) WT, H148Y, C45A, and H148A variants purified from *E. coli*. Bovine serum albumin (BSA) was included as a reference for protein quantification. *D* and *E*, In vitro deubiquitination (DUB) assays using purified FLAG-OTUD4^FL^ WT and variants and K48-linked polyubiquitin chains (Ub_2-7_) as a substrate. Ubiquitin cleavage was assessed by western blotting (WB) over a time-course (*D*) or with increasing enzyme concentrations (*E*). *F*, Co-immunoprecipitation (IP) of FLAG-tagged OTUD4 variants or GFP control expressed in HEK293T cells, followed by WB for the interacting protein USP7. Gels in *A–C* are representative of at least two independent purifications. Data in *D–F* are representative of at least three independent experiments.

**Supplementary Figure S2. OTUD4 interaction with the TAK1 signalosome and antibody validation.** *A*, Co-IP of the TAK1 signalosome. HEK293T cells stably expressing HA-TAK1 were transfected with either an empty vector or the indicated OTUD4^FL^ WT and variants. HA-tagged proteins were immunoprecipitated (IP: HA), and the precipitates and input lysates were analyzed by WB for the indicated proteins. β-tubulin served as a loading control. Data are representative of at least three independent experiments. *B*, Validation of phospho-TAK1 (p-TAK1) antibody specificity. WT and OTUD4^-/-^ MEFs were stimulated with TNF (5 ng/mL) for the indicated times. Lysates were prepared with or without phosphatase inhibitors and then incubated at 37 °C for 1 h before analysis. The p-TAK1 signal was abolished in lysates lacking inhibitors (compare lanes 4 and 8), confirming antibody specificity. This validation was performed in two independent experiments with similar results.
