# Supplementary material for: The deubiquitinase OTUD4 suppresses TAK1 kinase–dependent NF-κB signaling and inflammation
Source: J Biol Chem. 2025 Oct 7;301(11):110784. doi: 10.1016/j.jbc.2025.110784 (PMC12607013; doi:10.1016/j.jbc.2025.110784)
Supplement: Supplementary Figure S1 [file mmc2.pdf]

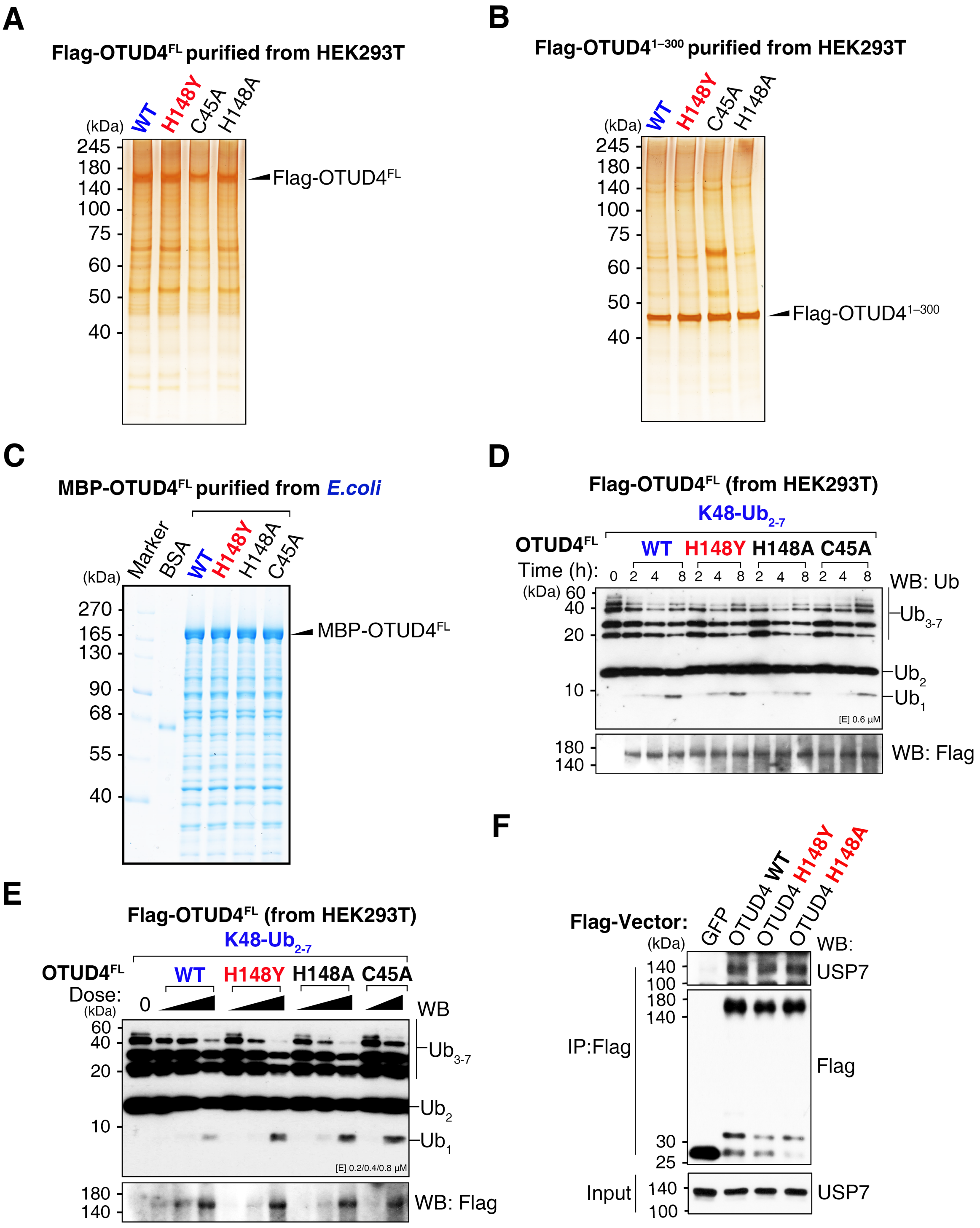
A
Flag-OTUD4FL purified from HEK293T
WT
H148Y
C45A
H148A
(kDa)
245
180
140
100
75
60
50
40
Flag-OTUD4FL
B
Flag-OTUD41–300 purified from HEK293T
WT
H148Y
C45A
H148A
(kDa)
245
180
140
100
75
60
50
40
Flag-OTUD41–300
C
MBP-OTUD4FL purified from E.coli
Marker
BSA
WT
H148Y
H148A
C45A
(kDa)
270
165
130
90
68
55
40
MBP-OTUD4FL
D
Flag-OTUD4FL (from HEK293T)
K48-Ub2-7
OTUD4FL
WT
H148Y
H148A
C45A
Time (h): 0 2 4 8 2 4 8 2 4 8 2 4 8
WB: Ub
(kDa) 60
40
20
10
Ub3-7
Ub2
Ub1
[E] 0.6 μM
180
140
WB: Flag
E
Flag-OTUD4FL (from HEK293T)
K48-Ub2-7
OTUD4FL
WT
H148Y
H148A
C45A
Dose:
0
WB
(kDa)
60
40
20
10
Ub3-7
Ub2
Ub1
[E] 0.2/0.4/0.8 μM
180
140
WB: Flag
F
Flag-Vector:
GFP
OTUD4 WT
OTUD4 H148Y
OTUD4 H148A
(kDa)
WB:
140
100
USP7
180
140
IP:Flag
Flag
30
25
Input
140
100
USP7
