# Supplementary figures and images for: The deubiquitinase OTUD4 suppresses TAK1 kinase–dependent NF-κB signaling and inflammation
Source: J Biol Chem. 2025 Oct 7;301(11):110784. doi: 10.1016/j.jbc.2025.110784 (PMC12607013; doi:10.1016/j.jbc.2025.110784)

**A**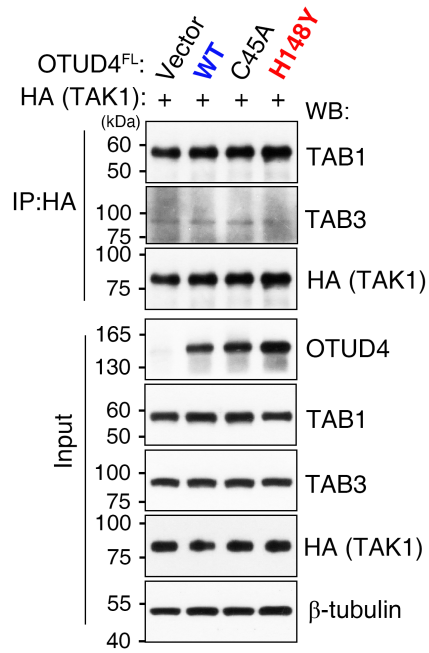**B**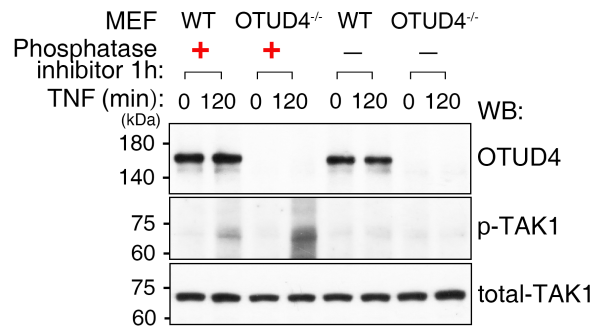

Supplement: Supplementary Figure S2 [file mmc3.pdf]
