# Supplementary material for: The deubiquitinase OTUD4 suppresses TAK1 kinase–dependent NF-κB signaling and inflammation
Source: J Biol Chem. 2025 Oct 7;301(11):110784. doi: 10.1016/j.jbc.2025.110784 (PMC12607013; doi:10.1016/j.jbc.2025.110784)
Supplement: Supplementary Table S1 Legends [file mmc4.pdf]

Table S1. Selected OTUD4-interacting proteins involved in the TAK1 signaling pathway

Gene Symbol	Swiss-Prot accession number	Molecular mass (kDa)	Total Peptides	Unique Peptides
OTUD4	Q01804	124.045	57	41
MAP3K7	O43318	67.196	9	9
TAB1	Q15750	54.644	15	16
TAB3	Q8N5C8	78.653	8	9
MYD88	Q99836	33.233	6	6
