# Supplementary material for: The deubiquitinase OTUD4 suppresses TAK1 kinase–dependent NF-κB signaling and inflammation
Source: J Biol Chem. 2025 Oct 7;301(11):110784. doi: 10.1016/j.jbc.2025.110784 (PMC12607013; doi:10.1016/j.jbc.2025.110784)
Supplement: Supplementary Table S1 [file mmc5.docx]

**Supplementary table legend**

**Supplementary Table S1**: **Identification of TAK1 signalosome components as OTUD4-interacting proteins.** Selected proteins were identified in a comprehensive proteomic screen as previously described (31).
